# Diagnostic Imaging Analysis of Humerus Bone Structure in Long‐Legged Buzzard (*Buteo rufinus*): Radiology and CT‐Scan Insights

**DOI:** 10.1002/vms3.71233

**Published:** 2026-10-04

**Authors:** Nafiseh Alinejad, Mohammad Reza Esmailinejad, Hemad Shafiei, Mohammadjavad Jahedi

**Affiliations:** ^1^ Islamic Azad University, Tehran, Iran; ^2^ Department of Clinical Science Faculty of Veterinary Medicine Shahid Bahonar University of Kerman Kerman Iran; ^3^ Shahid Bahonar University of Kerman Kerman Iran

**Keywords:** avian, *Buteo rufinus*, CT‐scan, diagnostic imaging, humerus, long‐legged buzzard, radiology

## Abstract

**Background:**

Advanced imaging techniques, particularly computed tomography (CT), are revolutionising veterinary diagnostics by providing detailed structural insights essential for accurate diagnosis and treatment planning.

**Objectives:**

This study aimed to compare the efficacy of CT imaging and digital radiography in the diagnosis and treatment of raptors, with the goal of identifying the more precise and efficient imaging technique for clinical management. By evaluating these methods, the research seeks to optimise diagnostic accuracy and treatment planning, ultimately improving rehabilitation outcomes for birds of prey requiring high‐precision care.

**Methods:**

This study evaluated the humerus bone structure of 10 adult Long‐legged Buzzard (Buteo rufinus) individuals using digital radiology and CT imaging. Birds were sedated to minimise stress, and both left and right humerus structures were examined. Humerus indices, including cranio‐caudal diameter, medullary cavity diameter, cortical thickness and bone density, were measured to compare the effectiveness of the two modalities.

**Results:**

Results of this study revealed significant differences in humerus indices between radiology and CT techniques (*P* < 0.05), with CT providing more accurate and detailed measurements. CT excelled in visualising internal structures, such as the cortex and medullary cavity, offering superior diagnostic clarity. No significant differences were observed between left and right humerus indices (*P* > 0.05), indicating consistent bilateral symmetry.

**Conclusions:**

The study concludes that CT imaging is superior to digital radiology for assessing humerus bone structure in the long‐legged buzzard, providing precise information crucial for managing humeral injuries. These findings underscore the clinical utility of CT in avian veterinary medicine, particularly for raptors and recommend This technique helps surgeons prepare the necessary equipment more accurately and use more precise methods, ultimately increasing the success rate of surgeries.

## Graphical abstract

This study utilised both computed tomography (CT) and digital radiography to analyse the humerus bone structure in the long‐legged buzzard (*Buteo rufinus*). The results highlight the value of these imaging techniques for detailed anatomical assessment and improved understanding of avian skeletal structure in veterinary diagnostics

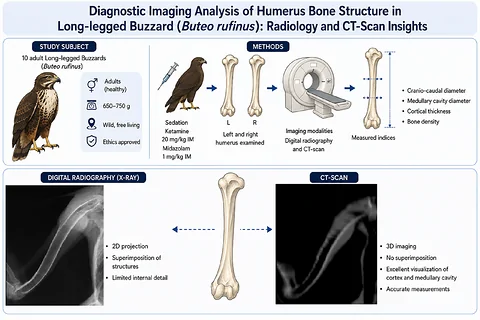

## 1 Introduction

The long‐legged buzzard (*Buteo rufinus*), a member of the family Accipitridae, exhibits a body length ranging from 50 to 65 cm, with a wingspan of 126 to 148 cm (Khaleghizadeh et al. 2020). This species, which closely resembles the common buzzard (*Buteo buteo*), displays a range of plumage colours from reddish‐brown to dark brown. Males and females share similar physical characteristics, with no noticeable seasonal variations in appearance (Khaleghizadeh et al. 2020). Long‐legged buzzards typically inhabit dry and semi‐dry steppes, grasslands, desert margins, hills and mountains. As apex predators, these birds serve as crucial indicators of local biodiversity (Khaleghizadeh et al. 2020).

Trauma‐induced fractures are the most frequent cause of admission for birds of prey to veterinary clinics, with common fractures occurring in the humerus, radius and ulna (Vergneau‐Grosset et al. 2019). Fractures of the femur, while less common, also occur. Due to their significant size and specialised flight mechanics, birds of prey are often among the most common species requiring fracture treatment in veterinary centres (Molina‐López et al. 2011). Consequently, evaluating bone structure integrity and identifying fractures in these birds is vital for effective treatment, surgery and rehabilitation following traumatic injuries, which are the primary reason for their referral to veterinary care centres (Molina‐López et al. 2011; Sönmez and Ergen 2023).

Diagnostic imaging is essential for evaluating bone structures and planning interventions in injured birds of prey. Advances in techniques such as digital radiography and, particularly, computed tomography (CT) have greatly improved diagnostic accuracy in avian and exotic animal medicine (Silva et al. 2020; Ricciardi et al. 2019). Radiographs remain invaluable for assessing the musculoskeletal system, detecting fractures and identifying soft tissue injuries (Hanna et al. 2021). However, conventional radiography is limited by the superimposition of anatomical structures. CT imaging overcomes this limitation by providing clear, detailed, three‐dimensional visualisation of anatomical features without overlap, offering superior diagnostic clarity for evaluating pathological changes (Krautwald‐Junghanns et al. 1998; EsmailiNejad et al. 2019).

Birds possess unique skeletal adaptations, most notably bone pneumatisation, in which air‐filled cavities within bones such as the humerus and femur connect to the respiratory system (Cooper 2002). This adaptation reduces skeletal weight, facilitating flight and aiding in respiration and air humidification, but it also increases bone fragility. Pneumatic bones are more prone to shattering or fragmenting upon impact, and their distal extremities lack substantial soft tissue protection, making them susceptible to open, comminuted fractures that complicate repair. Moreover, fractures of pneumatic bones, particularly the femur, may cause respiratory complications when open (Bennett and Kuzma 1992).

Despite the clinical importance of fracture diagnosis in raptors, few studies have compared the effectiveness of radiography and computed tomography (CT) in evaluating the humerus of the long‐legged buzzard. In this context, there is an increasing need for a thorough understanding of normal anatomy in healthy animals through the use of diagnostic imaging. This knowledge would enhance the ability to accurately identify and interpret pathological conditions in the long‐legged buzzard.

## 2 Materials and Methods

### 2.1 Animals

In Kerman province, a protected wildlife rehabilitation centre receives injured birds referred by environmental guards for treatment. Ten mature long‐legged buzzards (*Buteo rufinus*), each weighing between 650 and 750 g, were selected for this study.

In this study, the sex and precise age of long‐legged buzzards (*Buteo rufinus*) could not be determined. The species’ monomorphic nature prevents sex identification based on external morphology. Conducted outside the breeding season, the study lacked observable reproductive behaviours, and radiographic imaging showed no active gonads. Regarding age, all birds were examined by an avian specialist and confirmed to be mature based on physical characteristics. However, due to the absence of reliable indicators for precise age determination, only their maturity could be noted, and their exact age remained undetermined.

All birds were wild animals under medical care at the Kerman Rehabilitation Center, referred by the Environmental Protection Department of Kerman. The animal ethics committee of **** approved all of the stages of the study (NO. ****). Prior to the study, each bird underwent a physical examination to exclude health complications that could influence the results. Live body weight was measured individually using a digital balance, and anaesthetic doses were calculated accordingly. Birds were then sedated with a combination of ketamine (20 mg/kg) and diazepam (1 mg/kg) administered intramuscularly to minimise stress and movement during imaging. Following sedation, digital radiography and computed tomography (CT) were performed on the whole body, followed by detailed analysis of the wings.

### 2.2 Diagnostic Imaging Protocol

#### 2.2.1 Radiography

Two standard orthogonal views were obtained for each wing (medio‐lateral and ventro‐dorsal projections from the coelomic cavity), utilising a Direct Digital Radiography system (a fix x‐ray generator, GAMI, DR model, Milan, Italy). The imaging parameters were set at 60 kVp and 2.5 mAs, with a source‐to‐detector distance of 90 cm. The wings were positioned laterally, and the birds were placed in dorsal recumbency with their wings extended for proper positioning. All imaging was performed by a single individual (CLM). Images were stored in a picture‐archiving and communication system (PACS) for further analysis.

#### 2.2.2 Computed Tomography

All CT imaging was conducted using a 16‐slice scanner (NeuViz 16 Essence, multi‐slice ultra‐fast CT scanner, Neusoft, Shenyang, China). The animals were positioned in dorsal recumbency, and their wings and legs were fixed using microporous surgical tape to prevent movement during the scans. All scans were performed with settings of 120 kVp and 150 mAs, a rotation time of 1 s, a slice thickness of 1 mm and a pitch of 1. Following the scans, images were transmitted to the acquisition workstation and reconstructed into bone planes using specialised software (PACSPLUS, Medical standard workstation version 3.0).

#### 2.2.3 Measured Parameters

Eight parameters were assessed by one single observer in both radiology and CT imaging, as detailed: cranio‐caudal diameter of bone (measurement of bone diameter from the cranial cortex to the caudal cortex in the middle section of the diaphyseal region), diameter of the medullary cavity (measurement of the internal diameter of the bone's medullary cavity in the middle section of the diaphyseal region), thickness of the cortex (evaluation of the cortical bone's thickness in the middle section of the diaphyseal region), proximal to distal length (measurement of the total length of the humerus from the proximal to the distal end) (Figure 1), thickness of soft tissue (evaluation of the soft tissue in the proximal, middle and distal regions) (Figure 2), and bone density (analysis of bone opacity using pixel value and Hounsfield unit at the cortex, medullary cavity and surrounding soft tissue). These parameters were meticulously measured and recorded for comparative analysis between the two imaging modalities to evaluate their diagnostic accuracy and clinical utility (Figure 3).

**FIGURE 1:**
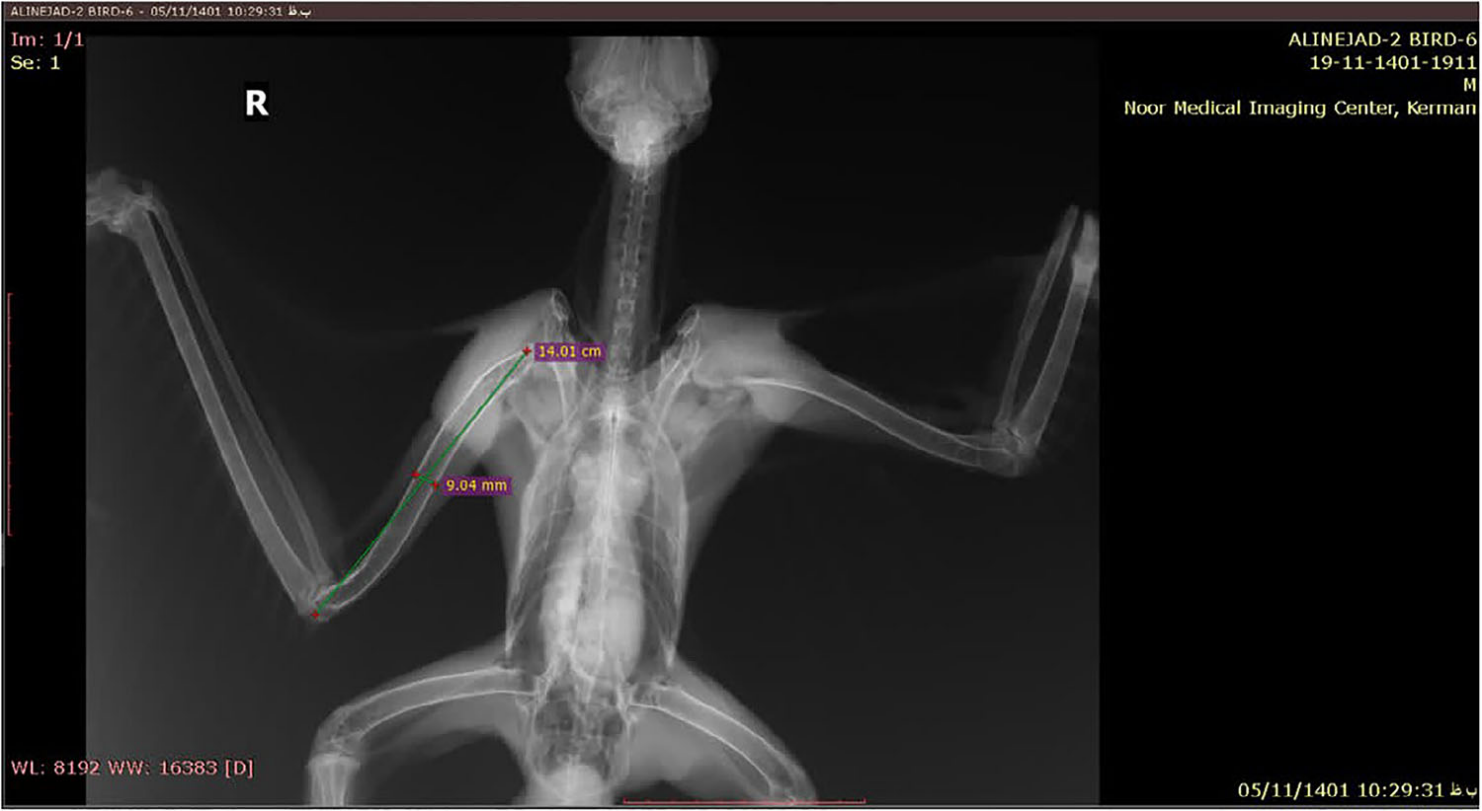
Medio‐lateral radiograph of the right wing of long‐legged buzzard, showing measurements of the humerus length from proximal to distal and the thickness of the humeral bone.

**FIGURE 2:**
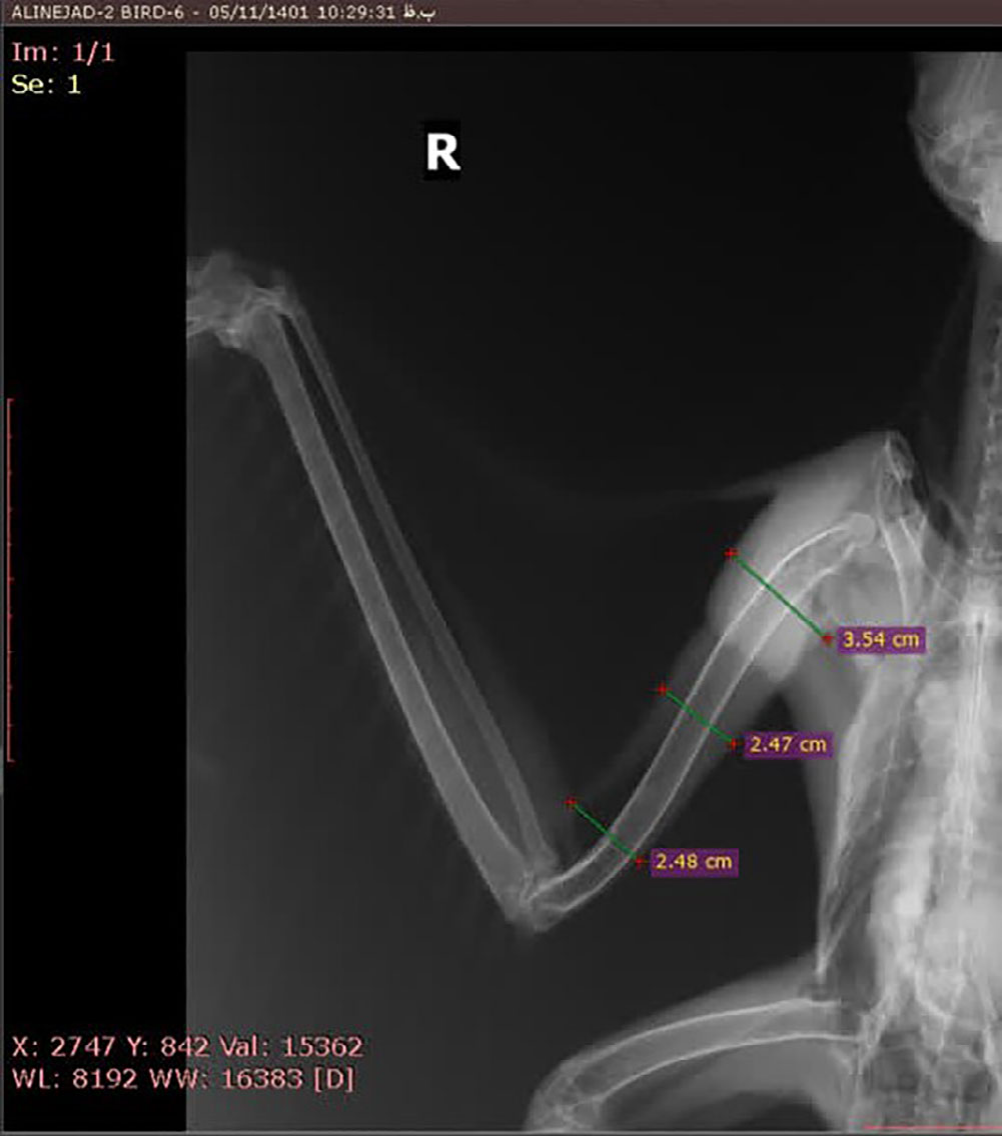
Medio‐lateral radiograph of the right wing of long‐legged buzzard, showing the thickness of soft tissue around the humerus measured at three sections: proximal, middle, and distal.

**FIGURE 3:**
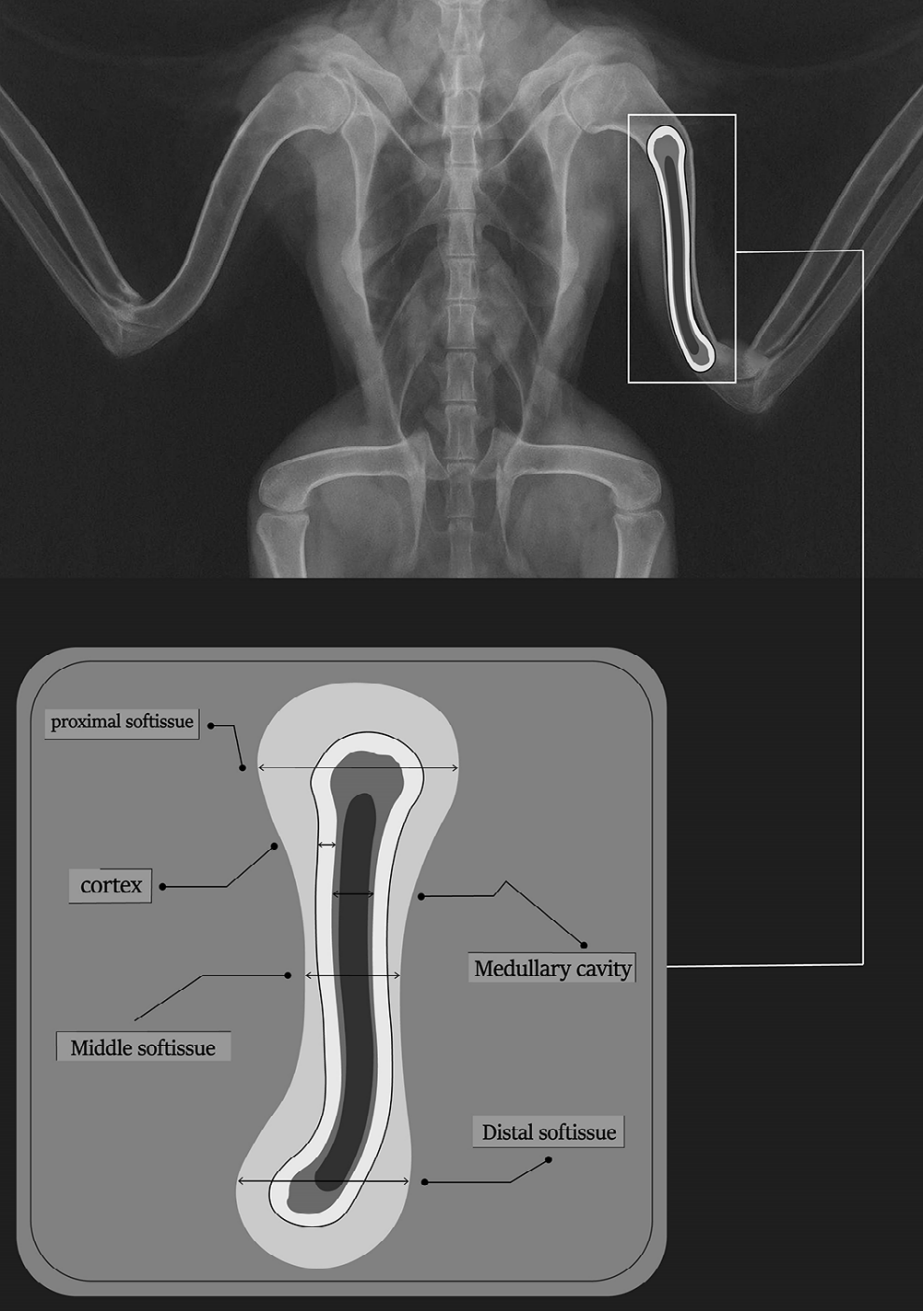
Ventro‐dorsal projection from the coelomic cavity that shows an annotated radiographic diagram illustrating the key components and anatomical regions assessed by radiographic evaluation.

#### 2.2.4 Statistics

All data collected in this study were analysed using SPSS statistical software version 21 (IBM Corporation, Armonk, NY, USA), and results were presented as mean ± standard deviation. For this analysis, independent t‐tests and paired t‐tests were employed. A significance level of 0.05 was established for this study.

## 3 Results

Table 1 presents the results of humerus bone parameter measurements obtained from digital radiography, while Table 2 displays the same parameters measured via CT scan for comparative purposes between the left and right wings.

**TABLE 1:** Examination of descriptive statistics in radiology technique for right and left wings.

Variable (mm)	Maximum		Minimum		Kurtosis		Skewness		SD		Mean	
	Right	Left	Right	Left	Right	Left	Right	Left	Right	Left	Right	Left
Cranio‐caudal diameter of bone (mm)	9.55	9.53	9.36	9.36	−1.928	−1.489	−0.268	− 0.312	0.074	0.063	9.467	9.459
Diameter of medullary cavity (mm)	7.52	7.50	7.12	7.13	−1.945	−1.892	0.008	0.035	0.161	0.151	7.324	7.316
Thickness of cortex (mm)	1.12	1.12	1.01	1.01	−1.649	−1.866	−0.332	−0.171	0.044	0.045	1.07	1.068
Proximal to distal length (mm)	140.9	140.8	140.5	140.06	−0.598	−0.604	−0.244	−1.916	0.134	0.227	140.74	140.626
Thickness of soft tissue in proximal part (mm)	39.17	39.15	37.12	37.11	−1.839	−1.818	−0.449	−0.463	0.928	0.914	38.339	38.328
Thickness of soft tissue in middle part (mm)	24.61	24.59	23.89	23.90	−1.847	−1.733	−0.378	−0.404	0.298	0.274	24.288	24.276
Thickness of soft tissue in distal part (mm)	20.05	20.02	19.62	19.63	−1.846	−1.63	−0.301	−0.347	0.167	0.150	19.872	19.866
Cortex value (pixel value)	8692	8693	7655	7785	5.371	5.639	−2.222	−2.266	309.3	270.85	8449.2	8485.7
Medullary cavity value (pixel value)	12219	12189	11496	11632	−1.782	−2.052	−0.211	−0.361	275.411	235.65	11886.9	11883.4
Soft tissue value (pixel value)	10092	10463	9243	9286	1.859	1.004	−1.407	−1.480	260.081	325.672	9828.7	9840.1

**TABLE 2:** Measurement of humeral indices in the CT‐scan method.

Variable	Maximum		Minimum		Kurtosis		Skewness		SD		Mean	
	Right	Left	Right	Left	Right	Left	Right	Left	Right	Left	Right	Left
Cranio‐caudal diameter of bone (mm)	9.46	9.46	9.32	9.34	−1.194	−1.197	−0.662	−0.666	0.051	0.051	9.406	9.407
Diameter of medullary cavity (mm)	5.83	5.83	5.42	5.51	0.222	0.226	−1.003	−1.003	0.135	0.138	5.678	5.679
Thickness of cortex (mm)	1.96	1.97	1.81	1.89	−0.208	−0.209	0.933	0.935	0.051	0.054	1.8622	1.8625
Proximal to distal length (mm)	134.65	134.75	131.23	131.15	−1.883	−1.883	−0.23	−0.23	1.295	1.297	132.845	132.841
Thickness of soft tissue in proximal part (mm)	38.40	38.20	35.40	36.30	1.955	1.959	−0.653	−0.653	0.928	0.914	37.19	37.29
Thickness of soft tissue in middle part (mm)	22.46	22.67	20.81	20.96	−0.766	−0.768	−0.29	−0.27	0.557	0.557	21.722	21.725
Thickness of soft tissue in distal part (mm)	19.71	19.73	17.98	18.02	−1.132	−1.135	0.074	0.076	0.594	0.594	18.846	18.849
Cortex value (HU)	863	867	783	786	−2.226	−2.229	0.095	0.094	35.299	35.307	822.3	822.7
Medullary cavity value (HU)	−739	− 735	−873	− 876	−1.867	−1.869	−0.224	−0.226	50.491	50.496	−805.3	− 806.3
Soft tissue value (HU)	73	75	59	62	−1.665	−1.661	0.101	0.101	5.522	5.226	65.6	66.6

The comparison between digital radiography and CT scan imaging revealed significant differences in their effectiveness for assessing the humerus in *Buteo rufinus* (Table 3). Although radiography provided an overall view of the bone structure, the CT scan provided more precise measurements of internal features, such as the thickness of the cortex and the diameter of the medullary cavity. However, no statistically significant difference was found in the cranio‐caudal diameter of the bone between digital radiography and CT scan techniques (P > 0.05). Nonetheless, the CT scan consistently provided more precise measurements than radiography and demonstrated superiority in distinguishing between the cortex and medullary cavity, with significant statistical differences (P < 0.05).

**TABLE 3:** Comparison of mean humeral features in CT‐scan and radiology for right and left wings.

Group	Radiology (Mean ± SD)		CT‐Scan (Mean ± SD)		Test statistic (degrees of freedom)		*p*‐value**	
	Right	Left	Right	Left	Right	Left	Right	Left
Cranio‐caudal diameter of bone (mm)	9.47 ± 0.02	9.46 ± 0.02	9.41 ± 0.02	9.40 ± 0.01	2.12 (18)	2.37 (18)	0.047 8	0.029 3
Diameter of medullary cavity (mm)	7.32 ± 0.05	7.32 ± 0.05	5.67 ± 0.04	5.66 ± 0.05	24.76 (18)	25.03 (18)	<0.00 1	<0.00 1
Thickness of cortex (mm)	1.07 ± 0.01	1.07 ± 0.01	1.86 ± 0.02	1.86 ± 0.02	−35.83 (17)	−35.12 (18)	<0.00 1	<0.00 1
Proximal to distal length (mm)	140.74 ± 0.04	140.63 ± 0.07	132.84 ± 0.41	133.30 ± 0.34	19.17 (18)	21.18 (18)	<0.00 1	<0.00 1
Thickness of soft tissue in proximal part (mm)	38.34 ± 0.92	38.33 ± 0.91	37.19 ± 0.83	37.29 ± 0.74	4.95 (18)	2.94 (18)	<0.00 1	<0.00 1
Thickness of soft tissue in middle part (mm)	24.29 ± 0.09	24.28 ± 0.09	21.72 ± 0.18	21.83 ± 0.16	12.82 (18)	13.53 (18)	<0.00 1	<0.00 1
Thickness of soft tissue in distal part (mm)	19.87 ± 0.05	19.87 ± 0.05	18.85 ± 0.19	18.93 ± 0.16	5.25 (18)	5.72 (18)	<0.00 1	<0.00 1
Cortex value (pixel, HU)	8449.2 ± 97.84	8485.7 ± 85.65	822.3 ± 11.16	817 ± 10.6	*	*	*	*
Medullary cavity value (pixel, HU)	11886.9 ± 87.09	11883.4 ± 74.52	−805.3 ± 15.97	−820 ± 11.35	*	*	*	*
Soft tissue value (pixel, HU)	9828.7 ± 82.24	9840.1 ± 102.97	65.6 ± 1.74	62.5 ± 1.9	*	*	*	*

The cranio‐caudal diameter of the bone measured 9.40 ± 0.05 mm with a CT scan and 9.46 ± 0.05 mm with digital radiography, highlighting a negligible difference between the two measurement methods. Similarly, the diameter of the medullary cavity averaged 5.67 ± 0.13 mm with the CT scan and 7.32 ± 0.05 mm with digital radiography, demonstrating that the CT scan provided a more distinct view of the medullary cavity. In terms of cortex analysis, the CT scan reliably visualised the cortical thickness, which averaged 1.86 ± 0.05 mm, whereas radiography measured it as 1.06 ± 0.01 mm (Figure 4).

**FIGURE 4:**
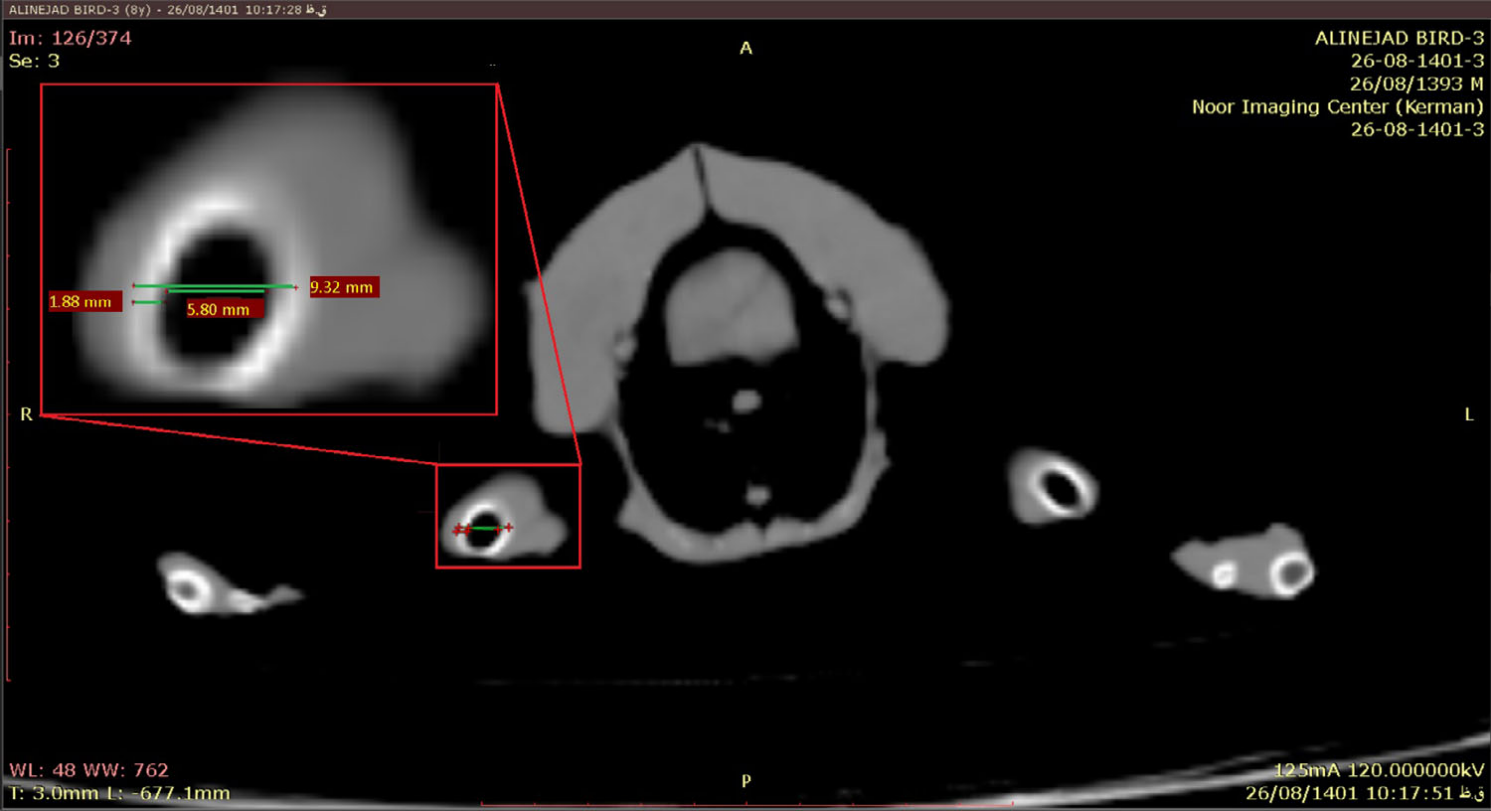
Transverse plane CT scan of the coelomic cavity and both wings of long‐legged buzzard, showing measurements of bone diameter, cortical thickness and medullary cavity thickness.

As the superior technique in this study, the CT scan indicated that there was no statistically significant difference between the left and right humerus for the evaluated parameters (*P* > 0.05) (Table 4). Key measurements of the humeral structure were consistent across specimens. The average proximal to distal length was measured as follows: digital radiography: 140.68 ± 0.05 mm, CT scan: 132.84 ± 0.05 mm, with no significant variation between the left and right sides in each technique (*P* > 0.05).

**TABLE 4:** Examination of mean differences in humeral features in the CT scan technique for the right and left wings.

Group	Right wing Mean ± SD	Left wing Mean ± SD	Test statistic (degrees of freedom)	*p*‐value
Cranio‐caudal diameter of bone (mm)	9.406 ± 0.05	9.40 ± 0.04	0.231 (18)	0.820
Diameter of medullary cavity (mm)	5.678 ± 0.13	5.65 ± 0.14	0.335 (18)	0.742
Thickness of cortex (mm)	1.8622 ± 0.05	1.87 ± 0.05	−0.351 (17)	0.73
Proximal to distal length (mm)	132.84 ± 1.29	133.30 ± 1.06	−0.860 (18)	0.401
Thickness of soft tissue in proximal part (mm)	37.19 ± 0.83	37.29 ± 0.74	−0.28 (18)	0.781
Thickness of soft tissue in middle part (mm)	21.72 ± 0.55	21.83 ± 0.50	−0.460 (18)	0.651
Thickness of soft tissue in distal part (mm)	18.84 ± 0.59	18.92 ± 0.49	−0.339 (18)	0.738
Cortex value (HU)	822.3 ± 35.29	817 ± 33.52	0.344 (18)	0.735
Medullary cavity value (HU)	−805.3 ± 50.49	−820.4 ± 35.90	0.771 (18)	0.451
Soft tissue value (HU)	65.6 ± 5.52	62.5 ± 6.02	1.200 (18)	0.246

Measurements of soft tissue thickness demonstrated variability along the length of the bone. The mean thicknesses were as follows: proximally, 38.33 ± 0.05 mm for digital radiography and 37.24 ± 0.05 mm for CT scan (Figure 5); at the midsection, 24.28 ± 0.05 mm for digital radiography and 21.72 ± 0.05 mm for CT scan; and distally, 19.86 ± 0.05 mm for digital radiography and 18.84 ± 0.05 mm for CT scan.

**FIGURE 5:**
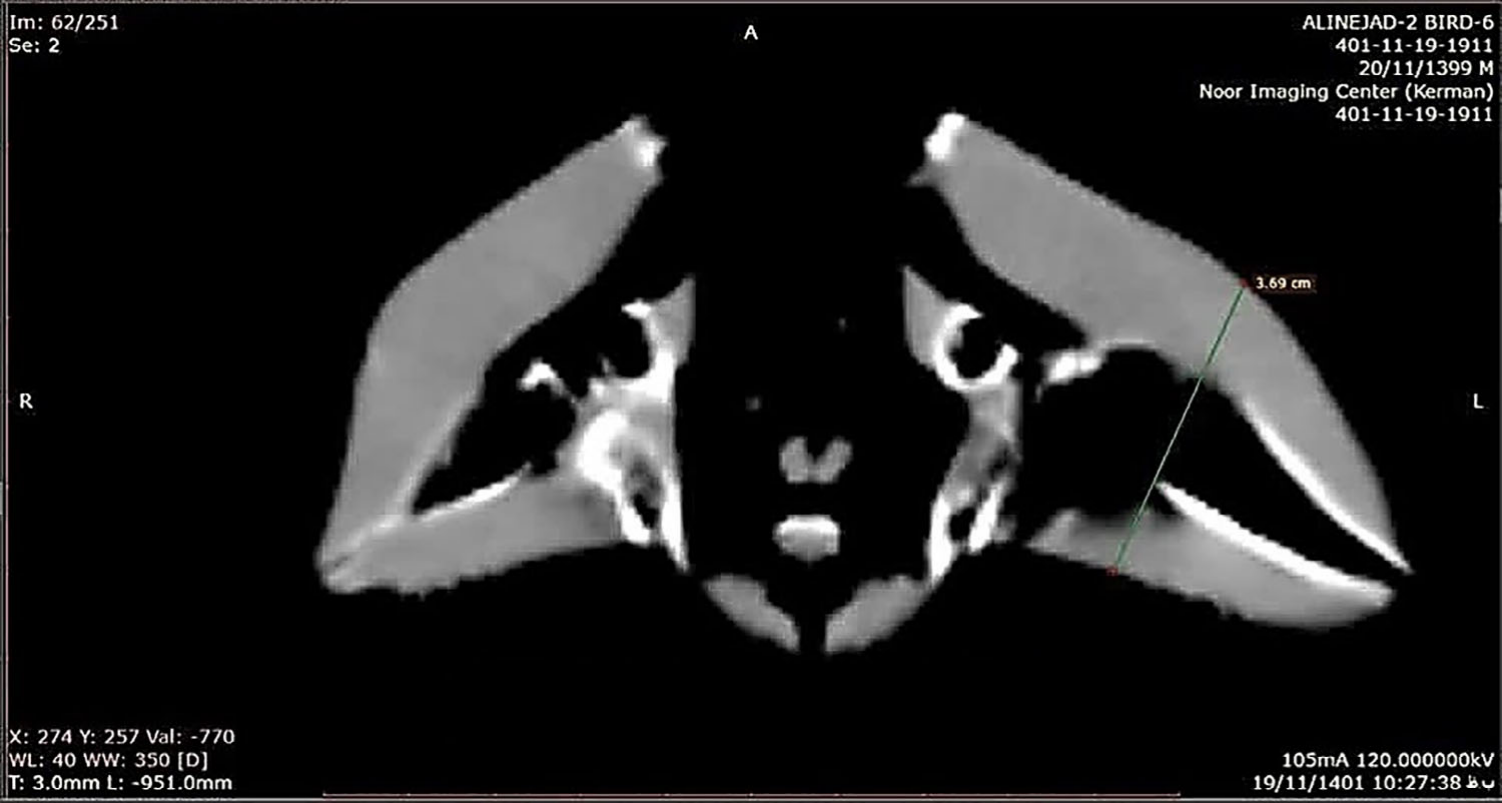
Transverse plane CT scan of long‐legged buzzard showing the thickness of soft tissue in the proximal part of the left humerus.

The analysis of pixel values in the cortex (right wing: 8449.2, left wing: 8485.7), medullary cavity (right wing: 11886.9, left wing: 11883.4) and surrounding soft tissues (right wing: 9828.7, left wing: 9840.1) demonstrated comparable results. Similarly, the evaluation of Hounsfield units (HU) in the cortex (right wing: 822.3, left wing: 822.7) (Figure 6), medullary cavity (right wing: −805.3, left wing: −806.3) and surrounding soft tissues (right wing: 65.6, left wing: 66.6) revealed no notable variation between the right and left wings. The Hounsfield unit (HU) of 800 ± 50 indicated density variations reflecting the degree of pneumatisation.

**FIGURE 6:**
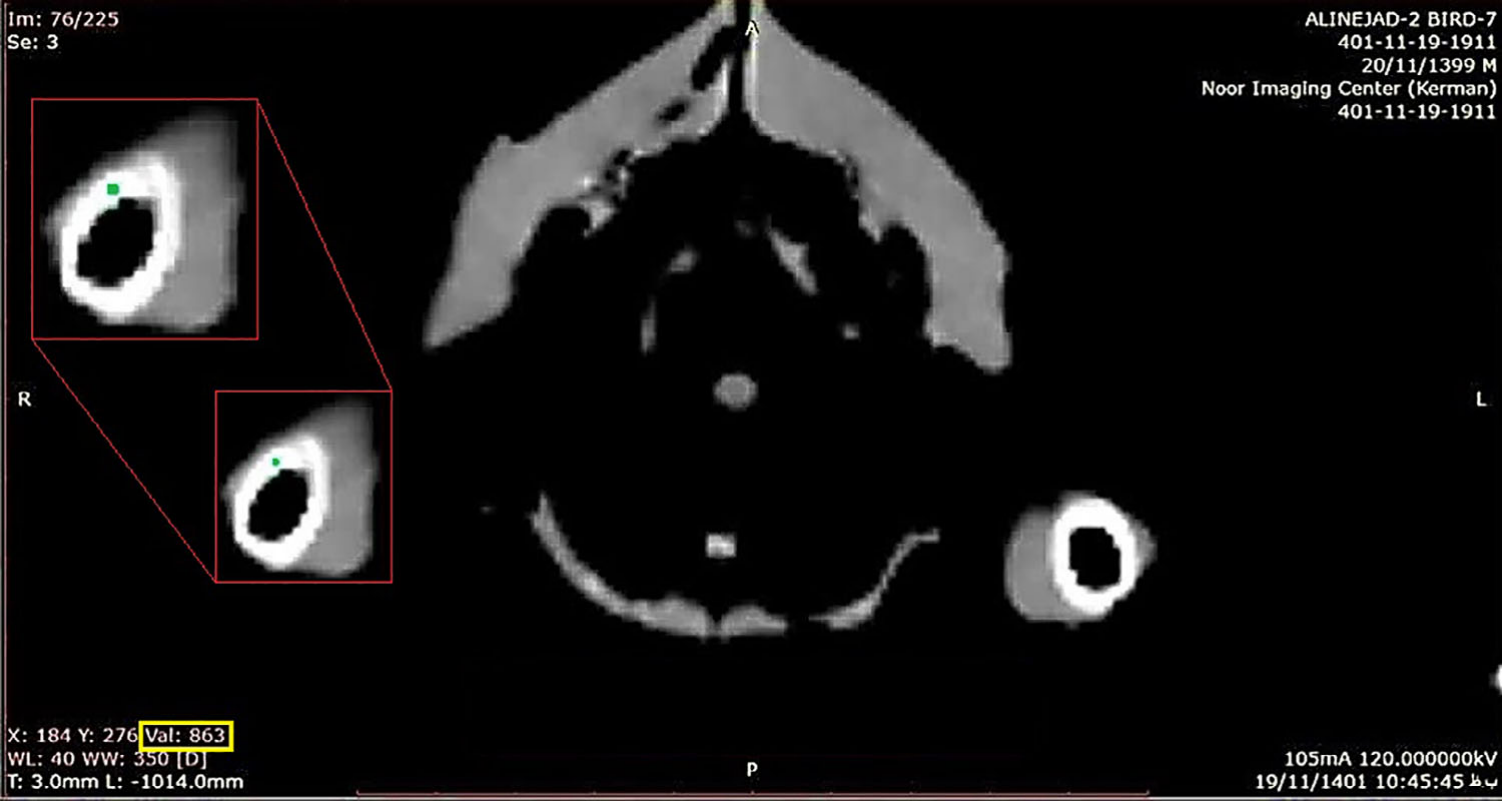
Transverse plane CT scan of long‐legged buzzard showing the hounsfield unit of the cortex in the right humerus.

## 4 Discussion

This study aimed to analyse the humeral bone structure in *Buteo rufinus* using advanced diagnostic imaging techniques, specifically digital radiography and computed tomography (CT). Ten mature specimens, referred to the Environmental Protection Department of Kerman, were examined to compare the effectiveness of these modalities in assessing the anatomical features of the humeral bone. Our findings demonstrate that CT scanning provides significantly superior diagnostic capabilities compared to digital radiography for evaluating avian bone structures, offering critical insights for veterinary treatment and conservation efforts.

The importance of diagnostic imaging, particularly CT scanning, in assessing avian skeletal structures is widely recognised in veterinary science. Numerous studies confirm the value of advanced imaging modalities for evaluating complex anatomical features and pathologies in birds, especially raptors (Cooper 2002; Grosso 2019; James 2017). CT scanning provides precise visualisation of bone density, trabecular patterns and anatomical details, enabling clinicians to assess fracture patterns and structural variations with remarkable clarity (Cooper 2002). Our findings strongly support these assertions, as CT imaging consistently showed better visualisation of internal bone structures, especially the cortex and medullary cavity, in the humerus of the long‐legged buzzard.

In a study by Bertuccelli et al., CT scanning was utilised to investigate factors predisposing raptors to humeral fractures, examining species such as the common buzzard (*Buteo buteo*) and the peregrine falcon (*Falco peregrinus*). Their findings demonstrated significant interspecies variation in bone density and structural dimensions, including cortex thickness and trabecular characteristics, suggesting that structural resilience in raptors is closely linked to anatomical adaptation to the demands of flight (Bertuccelli et al. 2021). Our findings align with these observations, as we documented significant differences in humeral parameters assessed through CT compared to radiography, highlighting CT's superior sensitivity in detecting subtle structural variations. Notably, our CT‐derived cortical thickness measurements (1.86 ± 0.05 mm) and medullary cavity diameters (5.67 ± 0.13 mm) provide species‐specific reference data for Buteo rufinus that complement the interspecies comparative framework established by Bertuccelli et al.

Recent advancements in high‐resolution CT scanning have enhanced avian anatomical studies by enabling precise assessments of inertial properties, essential for modelling flight dynamics and stability in birds of prey. Durston et al. employed calibrated CT scanning to measure the avian centre of mass and inertia tensor, contributing valuable data for biomechanical modelling and understanding the flight capabilities specific to raptors like *Buteo rufinus* (Durston et al. 2022). Our study enhances existing biomechanical research by offering detailed morphometric data on the humerus of *Buteo rufinus*. This data includes proximal‐distal length (132.84 ± 0.05 mm as measured by CT), cranio‐caudal diameter (9.40 ± 0.05 mm) and measurements of soft tissue thickness in various regions. These parameters provide essential baseline information for future biomechanical modelling and fracture risk assessments in this species.

The variability we observed in both cortical thickness and the diameter of the medullary cavity is consistent with findings from recent anatomical atlases that combine CT imaging with anatomical cross‐sections, such as those by Veladiano et al. These atlases provide valuable reference points for clinicians who are assessing species‐specific skeletal adaptations and pathologies (Veladiano et al. 2016). Our measurements add to this growing repository of reference data, showing that CT‐derived values offer greater precision than radiographic measurements. This is further supported by the significant differences observed between the two modalities (*P* < 0.05) for most parameters evaluated.

The use of quantitative micro‐computed tomography (micro CT) allows for detailed evaluations of cortical and trabecular structures crucial for managing avian fractures (Fajardo et al. 2007). Our findings show that CT imaging provides more accurate measurements of cortical thickness (1.86 ± 0.05 mm) and medullary cavity diameter (5.67 ± 0.13 mm) compared to radiography (1.06 ± 0.01 mm and 7.32 ± 0.05 mm, respectively). These differences indicate that radiography tends to overestimate medullary cavity diameter and underestimate cortical thickness due to structural superimposition and limited contrast. Fajardo et al. noted that while pneumaticity reduces cortical thickness, trabecular architecture compensates to maintain bone strength. Although our study did not specifically assess trabecular structures, the CT‐derived Hounsfield unit values for the cortex (around 822 HU), medullary cavity (around −805 HU) and soft tissues (around 66 HU) support these compensatory mechanisms.

Schneider further illustrated the efficacy of multi‐resolution CT scanning in the identification of species among mummified bird specimens, emphasising the versatility of CT scanning technology in the meticulous examination of various anatomical features. In clinical settings, multi‐resolution CT scanning offers a non‐invasive method for discerning structural details, which proves particularly advantageous for the assessment and treatment of fractures in veterinary practice (Schneide 2024). Our findings align with this perspective, as CT imaging demonstrated superior capabilities in distinguishing between cortical bone and medullary cavity, providing clinicians with more reliable information for surgical planning.

Similarly, recent studies on avian species, such as *Rupornis magnirostris* (the roadside hawk), have highlighted the utility of digital radiography and CT scanning for accurately visualising and assessing avian bone structures. Souza de Lima et al. conducted a detailed analysis using radiography and CT scanning to generate two‐dimensional and three‐dimensional models of the hawk's long bones, enabling precise identification of anatomical structures, including the humerus. In clinical practice, combining CT scanning with 3D digital modelling offers a robust tool for assessing fracture susceptibility and resilience under flight stresses, as observed in raptors like Buteo rufinus (de Lima et al. 2023). Our study corroborates these findings, demonstrating that CT imaging provides more precise measurements of humeral indices than digital radiography, with significant differences observed in medullary cavity diameter, cortical thickness and soft tissue thickness across all three anatomical regions examined (proximal, middle and distal). The integration of 3D rendering and radiographic analysis not only improves diagnostic precision but also has practical applications in surgical planning and post‐injury rehabilitation, supporting conservation efforts with minimally invasive intervention.

The aforementioned findings underscore the importance of integrating CT scanning, digital radiography, and quantitative CT scanning in avian anatomical studies. High‐resolution imaging facilitates detailed structural and functional analyses, offering a holistic view of skeletal health and biomechanical adaptations. The present study on *Buteo rufinus* emphasises the value of these techniques in veterinary care, aligning with broader anatomical studies that highlight the adaptability and resilience of avian skeletal structures under the demands of flight.

The integration of advanced imaging technologies (CT and digital radiography) has proven highly effective in examining and analysing the humerus structure in long‐legged buzzard. These techniques enable precise evaluation of key parameters: bone density, cortical thickness and trabecular patterns. Such measurements are vital for understanding skeletal integrity and biomechanics in these raptors.

Digital radiography yields valuable data on humeral indices and structural features, yet CT scanning is the superior diagnostic tool. Its capacity to generate high‐resolution, three‐dimensional views of bone architecture enables precise structural assessment. These detailed insights prove invaluable when designing targeted treatment plans and rehabilitation protocols for injured birds of prey, leading to better outcomes in care and conservation. This research demonstrates that advanced imaging techniques are crucial in avian veterinary medicine, significantly advancing raptor rehabilitation efforts.

## Author Contributions

**Nafiseh Alinejad**: software, funding acquisition, writing – review and editing. **Mohammad Reza Esmailinejad**: conceptualisation, methodology, data curation, investigation, validation, supervision, writing – review and editing. **Hemad Shafiei**: conceptualisation, investigation, validation, supervision, project administration, resources. **Mohammadjavad Jahedi**: formal analysis, visualisation, writing – original draft, writing – review and editing.

## Funding

The authors have nothing to report.

## Ethics Statement

The animal ethics committee of Shahid Bahor University of Kerman approved all of the stages of the study (NO. 1403101).

## Conflicts of Interest

The authors declare no conflicts of interest.

## Data Availability

The data that support the findings of this study are available from the corresponding author upon reasonable request.
